# Structural Insight Into Ryanodine Receptor Channelopathies

**DOI:** 10.3389/fphar.2022.897494

**Published:** 2022-05-23

**Authors:** Hadiatullah Hadiatullah, Zhao He, Zhiguang Yuchi

**Affiliations:** ^1^ Tianjin Key Laboratory for Modern Drug Delivery and High-Efficiency, Collaborative Innovation Center of Chemical Science and Engineering, School of Pharmaceutical Science and Technology, Tianjin University, Tianjin, China; ^2^ Department of Molecular Pharmacology, National Clinical Research Center for Cancer, Key Laboratory of Cancer Prevention and Therapy, Tianjin’s Clinical Research Center for Cancer, Tianjin Medical University Cancer Institute and Hospital, Tianjin, China

**Keywords:** ryanodine receptor, cryo-EM, x-ray crystallography, channelopathies, disease mutations

## Abstract

The ryanodine receptors (RyRs) are large cation-selective ligand-gated channels that are expressed in the sarcoplasmic reticulum (SR) membrane. They mediate the controlled release of Ca^2+^ from SR and play an important role in many cellular processes. The mutations in RyRs are associated with several skeletal muscle and cardiac conditions, including malignant hyperthermia (MH), central core disease (CCD), catecholaminergic polymorphic ventricular tachycardia (CPVT), and arrhythmogenic right ventricular dysplasia (ARVD). Recent breakthroughs in structural biology including cryo-electron microscopy (EM) and X-ray crystallography allowed the determination of a number of near-atomic structures of RyRs, including wildtype and mutant structures as well as the structures in complex with different modulating molecules. This allows us to comprehend the physiological gating and regulatory mechanisms of RyRs and the underlying pathological mechanisms of the disease-causing mutations. In this review, based on the insights gained from the available high-resolution structures of RyRs, we address several questions: 1) what are the gating mechanisms of different RyR isoforms; 2) how RyRs are regulated by multiple channel modulators, including ions, small molecules, and regulatory proteins; 3) how do disease-causing mutations affect the structure and function of RyRs; 4) how can these structural information aid in the diagnosis of the related diseases and the development of pharmacological therapies.

## 1 Introduction

The ryanodine receptor (RyR) is a giant allosteric ion channel that plays a crucial role in excitation-contraction (EC) coupling (Flucher and Franzini-Armstrong, 1996; Meissner, 2017; Ríos, 2018), neuron excitability (Albrecht et al., 2001; Bouchard et al., 2003; Arias-Cavieres et al., 2018), differentiation (Denda et al., 2012), and apoptosis (Tu et al., 2016; Bagur and Hajnóczky, 2017). RyR is mainly expressed on the membrane of endoplasmic reticulum (ER) or sarcoplasmic reticulum (SR) in muscle and mediates the release of calcium ions from ER/SR store. Mammals express three RyR isoforms (RyR1, RyR2, and RyR3), which share sequence identity of 63%–67% (Hakamata et al., 1992). RyR1 and RyR2 isoforms are predominantly found in skeletal and cardiac muscle, respectively (Takeshima et al., 1989; Otsu et al., 1990). However, the phrases “skeletal and cardiac isoforms” would be misleading since they are also expressed in a variety of cell types (Giannini et al., 1995). RyR3 was originally identified in the brain but later found to be distributed ubiquitously (Pessah et al., 1985). RyRs are also found in many lower organisms, which are similar in size and complexity to their mammalian counterparts. Non-mammalian vertebrates have two RyR isoforms, usually named as RyRα and RyRβ (OTTINI et al., 1996), whereas insects and other invertebrates, such as nematodes, sea urchin, fruit fly, and lobster, have only one isoform (Lanner et al., 2010).

More than 700 inherited genetic mutations of RyRs have been identified and associated with several life-threating diseases (Lehnart et al., 2008; Betzenhauser and Marks, 2010; Lanner et al., 2010). For example, RyR1 mutations have been primarily linked to malignant hyperthermia (MH) (FuJII et al., 1991; Gillard et al., 1991), central core disease (CCD) (Zhang et al., 1993), multi-minicore disease (MmD), congenital fiber type disproportion (CFTD), and centronuclear myopathy (CMD) (Treves et al., 2008; Amburgey et al., 2011; Amburgey et al., 2013; Snoeck et al., 2015; Jungbluth et al., 2018), and account for more than 30% of total congenital myopathy cases (Zvaritch et al., 2009; Zhou et al., 2013). RyR2 mutations have been associated with several cardiac conditions such as catecholaminergic polymorphic ventricular tachycardia (CPVT) (Laitinen et al., 2001; Itoh et al., 2021), sudden cardiac death (Blayney and Lai, 2009; Aiba et al., 2016), heart failure (HF) (Ran et al., 2010), and other cardiac arrhythmias (Priori et al., 2001). RyR3 mutations have been linked to neurodegenerative and cardiac diseases (Supnet et al., 2010; Yang et al., 2017; Gong et al., 2018), but the evidence is less clear. In addition, the mutations in all three isoforms are also associated with cancer in several clinical studies (Kobylewski et al., 2012; Lu et al., 2017; Schmitt et al., 2019; Xu et al., 2019; Liu et al., 2021). These dysfunctions highlight the key role of RyRs in human physiology and diseases. Understanding the impacts of these mutations on RyRs at the molecular level would not only provide important insights into the disease mechanisms but also lay a foundation for the development of therapeutic avenues to tackle these debilitating diseases.

**FIGURE 1:**
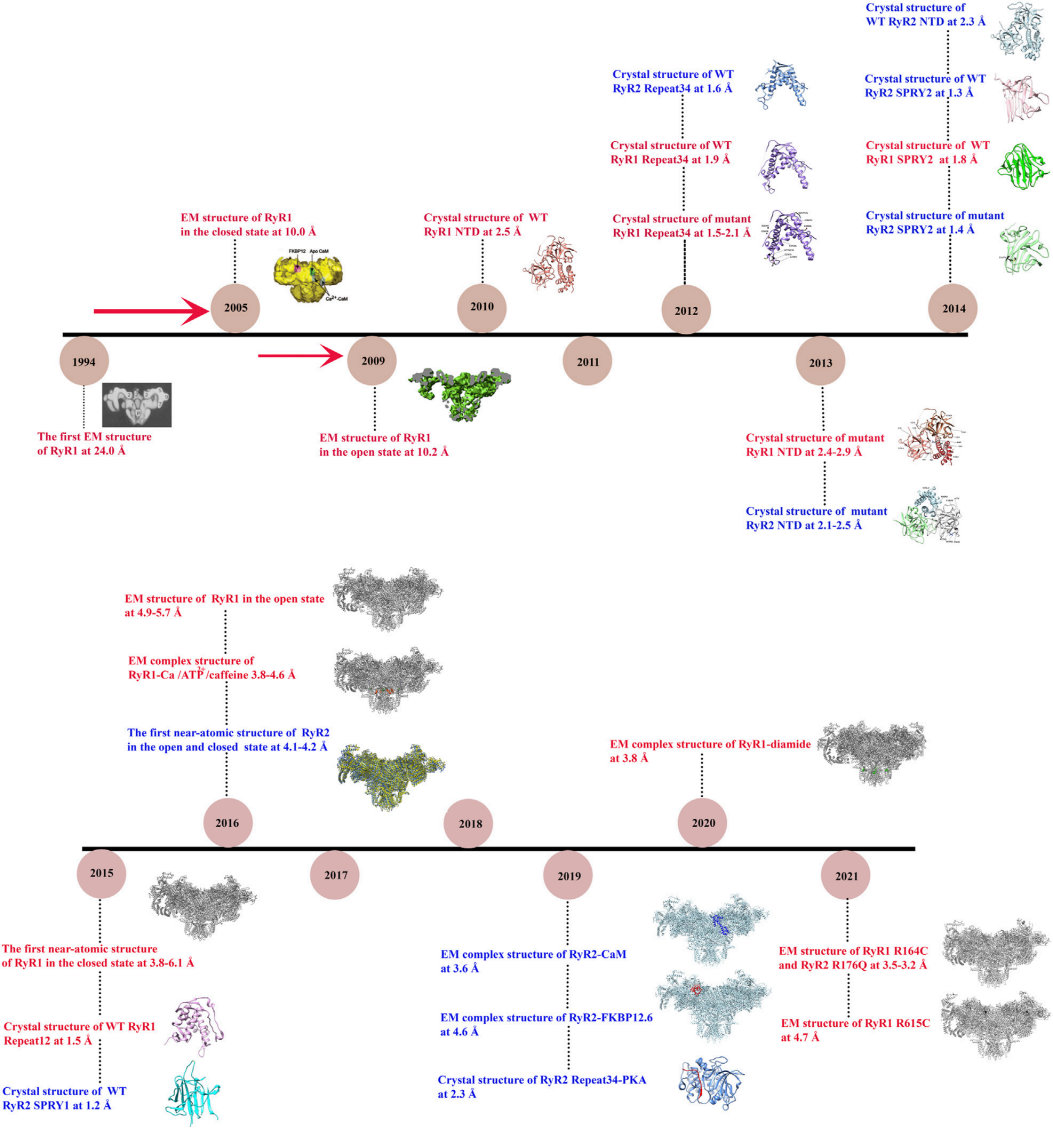
Timeline of significant discoveries/milestones of RyR structural studies. The structures of RyR were adapted from (Radermacher et al., 1994; Samsó et al., 2005; Samsó et al., 2009; Tung et al., 2010; Yuchi et al., 2012; Kimlicka et al., 2013a; Kimlicka et al., 2013b; Lau and Van Petegem, 2014; Efremov et al., 2015; Yan et al., 2015; Yuchi et al., 2015; Zalk et al., 2015; Bai et al., 2016; Wei et al., 2016; Chi et al., 2019; Gong et al., 2019; Haji-Ghassemi et al., 2019; Iyer et al., 2020; Ma et al., 2020; Woll et al., 2021).

Recently, thanks to revolutionary advances in structural biology techniques, several structures at near-atomic resolutions have been solved for both mammalian RyR1 (Efremov et al., 2015; Yan et al., 2015; Zalk et al., 2015; Bai et al., 2016; Wei et al., 2016) and RyR2 (Peng et al., 2016), revealing the overall domain organization and the gating mechanisms. In addition, the structures of RyRs with disease-causing mutations (Iyer et al., 2020; Woll et al., 2021) or small molecule modulators have also been reported (des Georges et al., 2016; Chi et al., 2019; Gong et al., 2019; Ma et al., 2020), elucidating the underlying disease and regulation mechanisms. Several good review papers have been published in recent years on the structure, function, and modulations of RyRs (Gong et al., 2021; Ogawa et al., 2021; Samurkas et al., 2022; Woll and Van Petegem, 2021). In this paper, we will first review the history of the structural studies of RyRs (summarized in Figure 1), and then mainly focus on the latest discoveries in RyR-related channelopathies from a structural point of view. Specifically, we will discuss the distribution of disease-causing mutations in the 3D structures of RyRs and how these mutations affect the structure and function of the channel. In addition, we will discuss the current developments in the pharmacology of RyRs.

## 2 Structures of RyRs Reveal the Overall Channel Architecture and Gating Mechanisms

### 2.1 Cryo-Electron Microscopy

**FIGURE 2:**
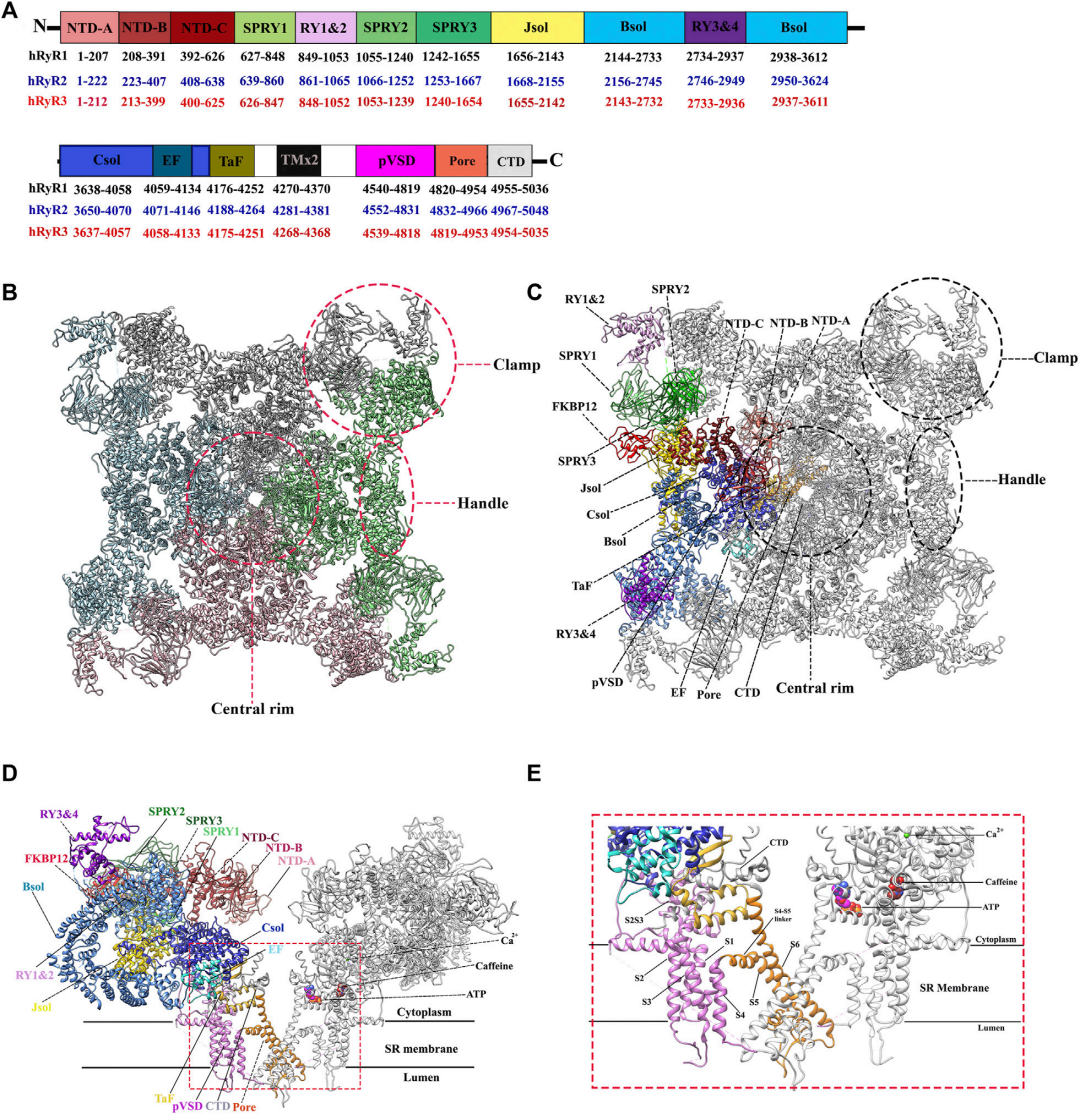
Overall structure and domain organization of RyR. **(A)** Schematic illustration of domain organization of three human RyR isoforms. The residue numbers of the domain boundaries are labeled. **(B)** The top view of the cryo-EM structure of rabbit RyR1 (PDB: 5TAL). Four protomers are colored in green, pink, cyan, and grey, respectively. Several distinct areas of the cytoplasmic region, including the clamp, the handle, and the central rim, are also labeled. **(C,D)** The top view **(C)** and the side view. **(D)** of the cryo-EM structure of rabbit RyR1 in complex with FKBP12, Ca^2+^, ATP, and caffeine (PDB: 5TAL). One of four protomers is colored by domain according to the schematic illustration in panel A. Two of the four protomers facing each other are shown for the side view. **(E)** A close-up view of the binding sites of Ca^2+^, ATP, and caffeine, and the transmembrane region.

In 1994, the first single-particle cryo-EM structure of the gigantic RyR isolated from rabbit skeletal muscle was solved by Wagenknecht group with a resolution of ∼40 Å (Radermacher et al., 1994). Since then, the resolution is gradually increased to ∼10 Å for the skeletal isoform with the efforts from several research groups during the next two decades (Samsó et al., 2005; Samsó et al., 2009; Ludtke and Serysheva, 2013). These medium-resolution structures revealed the overall shape of the channel as a giant tetrameric “mushroom” with four-fold symmetry, which contains ∼5,000 residues per monomer (Figure 2A) (Samsó et al., 2009). The symmetry axis is along the Ca^2+^-conducting pore of the channel and its transmembrane domain is like a classic voltage-gated ion channel such as Kv1.2 (Samsó et al., 2009). At this resolution, numerous distinct regions of the cytoplasmic part of RyR, including the clamp, the handle, and the central rim, could be resolved (Figure 2B), providing valuable information for the domain organization, although the exact corresponding sequences were still not clear.

**FIGURE 3:**
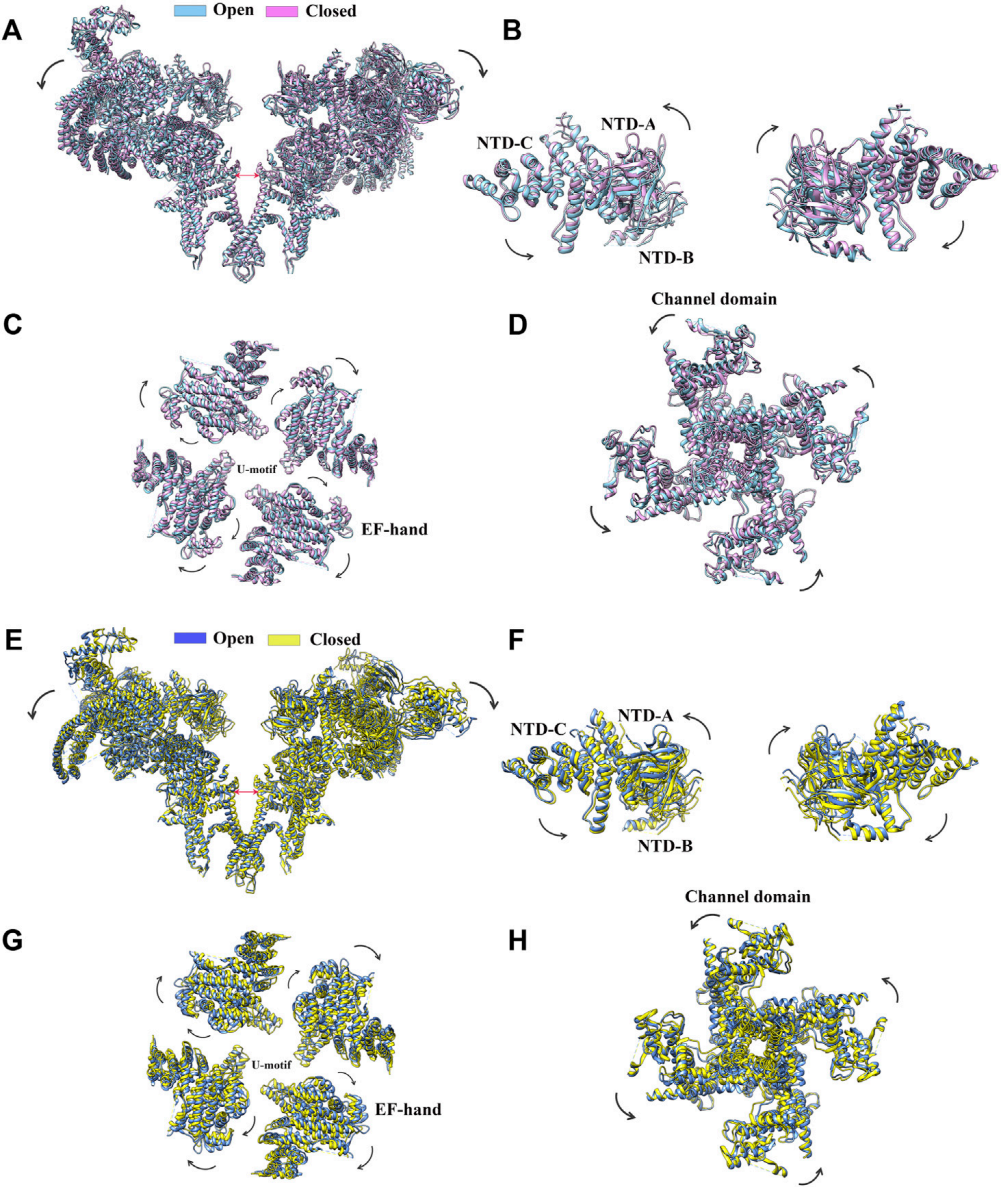
Conformational changes of RyR upon channel gating. **(A)** Superimposition of RyR1 in the closed state (magenta, PDB: 5TB0) and the open state (sky blue, PDB: 5TAL). The structural movements of individual domains of RyR1, such as the NTD. **(B)**, the central domain. **(C),** and the channel domain. **(D)** are shown in a zoomed-in view. **(E)** Superimposition of RyR2 in the closed state (yellow, PDB: 5GO9) and the open state (light blue, PDB: 5GOA). Two of the four protomers facing each other are shown. The structural movements of individual domains of RyR2, such as the NTD. **(F)** The central domain. **(G)** and the channel domain. **(H)** are shown in a zoomed-in view.

Since the early 2010s, the “resolution revolution” in cryo-EM has dramatically improved the quality of RyR structures. The cryo-EM structures of rabbit RyR1 have been solved by several groups with resolutions of 3.2–6.1 Å, representing different functional states, including the closed-state (Efremov et al., 2015; Yan et al., 2015; Zalk et al., 2015; des Georges et al., 2016), the open-state (Bai et al., 2016; des Georges et al., 2016; Wei et al., 2016), the primed-state (des Georges et al., 2016), and several ligand-bound states, such as in complex with Ca^2+^, ATP, caffeine, ryanodine (des Georges et al., 2016), calmodulin (CaM) (Gong et al., 2019; Ma et al., 2020), FK506-binding protein (FKBP) (Yan et al., 2015; des Georges et al., 2016; Peng et al., 2016; Chi et al., 2019; Gong et al., 2019), and diamide (Ma et al., 2020). With the improved resolution, a total of 20 individual domains from each protomer become discernible, including three N-terminal domains (NTDs) (NTD-A, NTD-B, and NTD-C), three SPRY domains (SPRY1, SPRY2, and SPRY3), three divergent regions (DR1, DR2, and DR3), two RYR repeat domains (Repeat12 and Repeat34), three solenoid (sol) domains (Bridging solenoid (Bsol), core solenoid [(Csol), and junctional solenoid (Jsol)], a shell-core linker peptide (SCLP) domain, an EF-hand domain (EF), a thumb and forefinger (TaF) domain, a pseudo voltage-sensor domain (pVSD), a channel pore domain (Pore), and a C-terminal domain (CTD) (Figures 2A,C). The last three domains form the transmembrane region enclosing a central Ca^2+^-conducting pore, whereas the other domains located in the cytoplasmic region mainly sense the signals from diverse ligands. The activity of RyR is regulated by a wide range of stimuli from both cytosolic and luminal sides, including ions (Ca^2+^, Mg^2+^, and Zn^2+^), proteins (CaM and FKBP12/12.6), and small molecules (ATP, caffeine, ryanodine, PCB95, and diamide insecticide) (Figures 2D,E) (Yan et al., 2015). The structural basis of channel gating and ligand-dependent activation has been established by comparing the open and closed structures of RyR1 (Figures 3A–D) (Bai et al., 2016; des Georges et al., 2016), which shows a coupled motion between the displacements of the cytoplasmic “O-ring” motif of the channel domain, the U-motif of the central domain, the central domain, and the dilation of the S6 helix bundle at the cytoplasmic side during pore opening (Figure 3C) (Bai et al., 2016). Des Georges et al. reported that either ATP, Ca^2+^, or caffeine alone promotes conformational changes in the cytoplasmic assembly, allowing it to reach a “primed” state without pore dilation. In contrast, the presence of all three activating regulators causes the pore dilation and conformational changes in the cytosolic assembly and local changes in the transmembrane domain (Figure 2E) des Georges et al., 2016).

Shortly after the determination of the rabbit RyR1 structure, Yan group solved the cryo-EM structures of porcine RyR2 in both the open and closed states (apo-RyR2) with a resolution of 4.2 and 4.4 Å, respectively (Figures 3E–H) (Peng et al., 2016). The comparison of the open and closed states of RyR2 shows relative movements within the cytoplasmic domains, leading to breathing motion of the periphery of the cytoplasmic region and the rotation of the central domain (Figure 3G). The central domain was shown to be the major mediator of conformational changes, integrating the conformational changes of the cytoplasmic domains and converting them into channel gating (Peng et al., 2016). Recently, complex structures of RyR2 with allosteric regulators Ca^2+^, PCB95, ATP, caffeine, FKBP12.6, and CaM were also determined in different configurations, gaining insight into the complex regulatory mechanisms of RyR2 (Chi et al., 2019; Gong et al., 2019). Aligning with their 70% sequence similarity, the overall structure and domain organization of RyR2 are almost identical to that of RyR1 (Yan et al., 2015), and the structural changes in the closed and open states of RyR2 are also largely similar to the ones in RyR1 (des Georges et al., 2016; Chi et al., 2019; Gong et al., 2019). However, many of the isoform-specific differences in function remain poorly defined, partially because that the non-conserved regions tend to be more flexible which are less resolved in the structures.

RyRs are known to form 2D lattice on ER membrane and their activity can be regulated by the crosstalk between neighboring RyR channels, or “coupled gating.” The potential of long-range allosteric gating presents a fascinating view of the impact of nano- and microdomains on intracellular calcium dynamics (Chen-Izu et al., 2006; Hiess et al., 2018). Therefore, in addition to the high-resolution structure of single isolated RyRs, the determination of the super-complex structure that can describe the structural linkage with neighboring RyRs is crucial for the understanding of the “coupled gating” mechanism. Using direct stochastic optical reconstruction (dSTORM) imaging several groups demonstrated that RyR2 may be clustered into a broad variety of group dimensions in ventricular cardiomyocytes, which designated as “super-clusters” (Hou et al., 2015; Munro et al., 2016). These super-clusters are groups of individual clusters close enough to each other that Ca^2+^ release from one cluster can cause Ca^2+^-induced Ca^2+^ release (CICR) from a neighboring cluster within the group (Macquaide et al., 2015). As clusters inside a super-cluster may be functionally connected, they are often referred to as calcium release units (CRUs). Along with inter-cluster spacing, the packing, or density, of RyR2 channels within individual clusters might influence their physiological Ca^2+^ handling. Reduced RyR2 channel density within a cluster increases the space between individual channels, reducing the possibility of RyR2–RyR2 interactions and thereby affecting coupled gating features and channel open probability (Walker et al., 2015). Recently, using a combination of line scan confocal imaging, dual-tilt electron tomography (ET), and dSTORM imaging, Asghari et al. (2020). reported that the arrangement of RyR2 super-complex is dynamic and can switch between different modes depending on the conditions of cellular environment, including the presence or absence of the regulatory proteins FKBP12 and FKBP12.6 as well as the post-translational modifications (PTMs) such as phosphorylation. Their findings prove that the long-range allosteric regulations by protein modulators or PTMs can change the size of RyR2 cluster and also the arrangement of RyR2 tetramers within clusters (Asghari et al., 2020). Advances in high-resolution techniques and approaches that can cover a broad range of dimensions, such as correlative light and electron microscopy (CLEM) that combines high-resolution structural data from cryo-electron tomography (cryo-ET) with spatiotemporal information from fluorescence light microscopy (FLM), would be necessary to solve the high-resolution structures of the super-complexes of RyRs, which would provide valuable information on the mechanism of “coupled gating.”

### 2.2 X-Ray Crystallography

While cryo-EM structures shed light on the overall domain organization of RyRs and conformational changes associated with channel gating, x-ray crystallography has been extensively used to reveal the structural details of RyR domains at atomic resolutions. Several high-resolution structures of RyR1, RyR2, and RyR3 domains were determined by X-ray crystallography, including whole NTDs of RyR1 (Tung et al., 2010; Kimlicka et al., 2013a) and RyR2 (Borko et al., 2014) and their individual subdomains (Amador et al., 2009; Lobo and Van Petegem, 2009; Amador et al., 2013), SPRY1 of RyR2 (Yuchi et al., 2015) and SPRY2s of RyR1 and RyR2 (Lau and Van Petegem, 2014; Yuchi et al., 2015), the Repeat12 of RyR1 (Yuchi et al., 2015), and the Repeat34 of RyR1, RyR2, and RyR3 (Table 1) (Yuchi et al., 2012; Yuchi et al., 2015).

**TABLE 1:** Structures of wild type and mutant RyRs mentioned in the paper.

Domain/isoform	Organism	WT/mutation	PDB	Experimental method	Resolution (Å)	References
RyR1-NTD	*Oryctolagus cuniculus*	WT	2XOA	X-ray Diffraction	2.05 Å	Tung et al. (2010)
RyR1-NTD	*Oryctolagus cuniculus*	L14R	4I7I	X-ray Diffraction	2.40–2.95 Å	Kimlicka et al. (2013a)
		G249R	4I1E			
		C36R	4I0Y			
		V219I	4I8M			
		I404M	4I2S			
		R45C	4I6I			
		D61N	4I3N			
		R402G	4I37			
RyR1-Repeat34	*Oryctolagus cuniculus*	WT	4ERT	X-ray Diffraction	1.95 Å	Yuchi et al. (2012)
RyR1-Repeat34	*Oryctolagus cuniculus*	R2939S	4ESU	X-ray Diffraction	1.59–2.19 Å	Yuchi et al. (2012)
		S2776M	4ETT			
		E2764K	4ETU			
RyR1-Repeat12	*Oryctolagus cuniculus*	WT	5C30	X-ray Diffraction	1.55 Å	Yuchi et al. (2015)
RyR1-SPRY2	*Oryctolagus cuniculus*	WT	4P9J	X-ray Diffraction	1.84 Å	Lau and Van Petegem, (2014)
Full-length RyR1	*Sus scrofa*	WT	6W1N	Cryo-EM	4.00 Å	Woll et al. (2021)
Full-length RyR1	*Sus scrofa*	R615C	6X34	Cryo-EM	4.70 Å	Woll et al. (2021)
Full-length RyR1	*Oryctolagus cuniculus*	R164C	6WOT	Cryo-EM	3.54 Å	Iyer et al. (2020)
Full-length RyR1	*Oryctolagus cuniculus*	Closed-state	5TB0	Cryo-EM	4.40 Å	des Georges et al. (2016)
Full-length RyR1	*Oryctolagus cuniculus*	Open-state	5TAL	Cryo-EM	4.40 Å	des Georges et al. (2016)
RyR2-NTD	*Homo sapiens*	WT	4JKQ	X-ray Diffraction	2.39 Å	Borko et al. (2014)
RyR2-NTD	*Mus musculus*	P164S	4KEI	X-ray Diffraction	2.14–2.55 Å	Kimlicka et al. (2013b)
		R169Q	4KEJ			
		R176Q	4KEK			
RyR2-NTD	*Mus musculus*	V186M; A77V	3IM6; 3IM7	X-ray Diffraction	1.70–2.21 Å	Lobo and Van Petegem, (2009)
RyR2-Repeat34	*Mus musculus*	WT	4ETV	X-ray Diffraction	1.65 Å	Yuchi et al. (2012)
RyR2-SPRY2	*Mus musculus*	WT	4P9I	X-ray Diffraction	1.34 Å	Lau and Van Petegem, (2014)
RyR2-SPRY2	*Mus musculus*	A1107M	4P9L	X-ray Diffraction	1.43 Å	Lau and Van Petegem, (2014)
RyR2-SPRY2	*Mus musculus*	P1124L	5VSN	X-ray Diffraction	1.43 Å	Alvarado et al. (2019)
RyR2-SPRY1	*Mus musculus*	WT	5C33	X-ray Diffraction	1.21 Å	Yuchi et al. (2015)
RyR2-SPRY1	*Mus musculus*	I784F	6J6L	X-ray Diffraction	1.21 Å	Touat-Hamici et al. (2021)
Full-length RyR2	*Homo sapiens*	WT	6WOV	Cryo-EM	5.10 Å	Iyer et al. (2020)
Full-length RyR2	*Homo sapiens*	R176Q	6WOU	Cryo-EM	3.27 Å	Iyer et al. (2020)
Full-length RyR2	Sus scrofa	Closed-state	5GO9	Cryo-EM	4.40 Å	Peng et al. (2016)
Full-length RyR2	Sus scrofa	Open-state	5GOA	Cryo-EM	4.20 Å	Peng et al. (2016)

### 2.2.1 NTD

**FIGURE 4:**
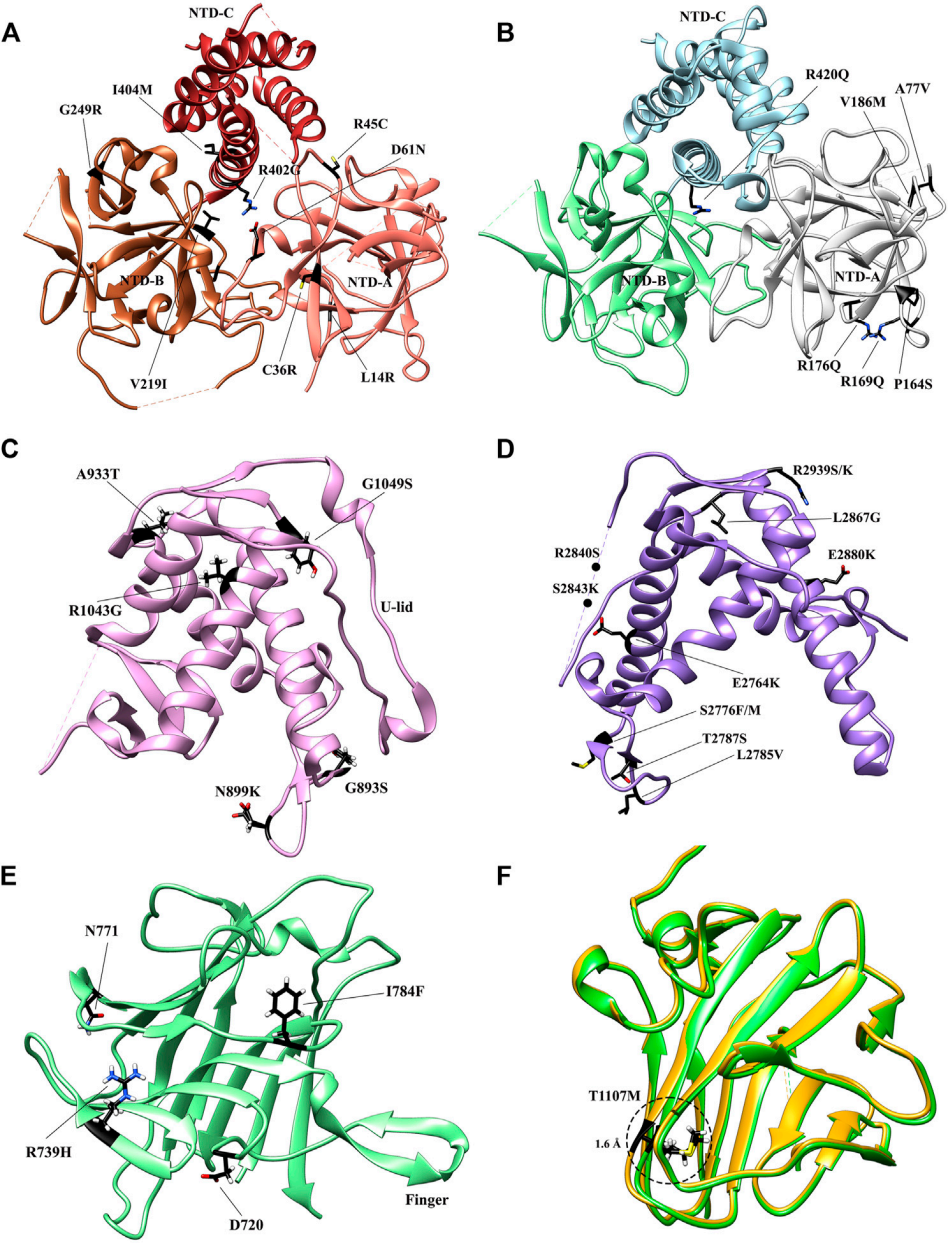
Crystal structures of the RyR domains with disease mutations mapped. **(A)** The crystal structure of the N-terminal domain of rabbit RyR1 (PDB: 2XOA). The individual subdomains are colored in sandy brown (NTD-A), sienna (NTD-B), and dark brown (NTD-C), respectively. **(B)** The crystal structure of the N-terminal domain of human RyR2 (PDB: 4JKQ). The individual subdomains are colored in grey (NTD-A), light green (NTD-B), and light blue (NTD-C), respectively. **(C)** The crystal structure of Repeat 34 domain of rabbit RyR1 (PDB: 4ERT). **(D)** The crystal structure of Repeat 12 domain of rabbit RyR1 (PDB: 5C30). **(E)** The crystal structure of SPRY1 domain of mouse RyR2 (PDB ID: 6J6L). **(F)**. The superposition of the crystal structures of WT SPRY2 (green) (PDB: 4P9I) and SPRY2 A1107M (golden) (PDB: 4P9L) in RyR2. The locations of individual disease-causing mutations are shown as black sticks and labelled.

Several reports have provided high-resolution details of the N-terminal domains (NTD) of RyR1 (Figure 4A) (Tung et al., 2010; Kimlicka et al., 2013a) and RyR2 (Figure 4B) (Borko et al., 2014). Based on the crystallographic studies of the N-terminal region of RyR, this region consists of three domains interacting with each other *via* a hydrophilic interface. NTD-A (residues 1–208 rabbit RyR1 numbering) and NTD-B (residues 209–392 rabbit RyR1 numbering) form β-trefoil domains, each containing 12 β-strands, while NTD-C (residues 393–627 rabbit RyR1 numbering) forms an armadillo repeat domain consisting of a bundle of five α-helices. These three domains are highly similar in sequence and structure to their counterparts of the inositol 1,4,5-triphosphate receptor (IP3R). NTD-B and NTD-C of IP3R form a ligand binding site for IP3, and the presence of NTD-A reduces the binding affinity of the ligand, hence named as ligand binding suppressor domain (Lin et al., 2011). In contrast, despite of the structural similarity, the ligand-binding property is not conserved in the RyR. According to the results of docking study, using several different RyR1 cryo-EM maps, the NTD is consistently docked in the center of the cytoplasmic region and forms a vestibule around the 4-fold symmetry axis (Tung et al., 2010). Albeit this position seems to be in contradiction with a GFP insertion study (Wang et al., 2007), the data can be aligned when the length of the linkers and the size of the insertion proteins are taken into account (Tung et al., 2010).

The overall structure of RyR2 NTD is similar to RyR1 (Figures 4A,B), but its NTD-A contains an additional α-helix, as revealed by an X-ray crystal/NMR hybrid structure (Amador et al., 2013). This mobile helix was not resolved in some other crystal structures (Lobo and Van Petegem, 2009; Kimlicka et al., 2013a; Borko et al., 2014), probably due to its high dynamics. According to the docking result, this α-helix is located at an interface with the pore region and might mediate the conformational changes between NTD and the pore (Amador et al., 2013).

Recently, the crystal structures of insect RyR NTD-A from two insect species, *Apis mellifera* and *Plutella xylostella,* have also been solved at the resolutions of 2.50 and 2.84 Å, respectively (Lin et al., 2018; Zhou et al., 2020). Several species-specific structural features were revealed at the inter-domain interfaces, which would alter conformation upon channel gating, making them excellent pest-specific insecticide-targeting sites.

### 2.2.2 Repeat12 and Repeat34

RyRs encode four tandem repeats existing in two pairs (Repeat12 and Repeat34). These domains are referred to as Repeat domains or RYR domains, and the Repeat34 domain is also named as the phosphorylation domain. Repeat12 (residues 850–1,054 rabbit RyR1 numbering), and Repeat34 (residues 2,735–2,938 rabbit RyR1 numbering), presumably evolved by gene-duplication, are ∼1,700 residues apart in sequence (Figure 2A). Because of tandem repeats, both domains have a pseudo two-fold symmetry.

The high-resolution crystal structures of Repeat34 (phosphorylation domain) have been solved for all three mammalian isoforms (Sharma et al., 2012; Yuchi et al., 2012; Yuchi et al., 2015). Repeat34 is positioned in the center of the RyR sequence and comprises a phosphorylation hot-spot loop that links two halves of the domain (Figure 2A). It exhibits a high pseudosymmetry and a prominent horseshoe shape (Figure 4C). Multiple phosphorylation sites have been reported in the Repeat34 domain of RyR1 (S2843) and RyR2 (S2808 and S2814), but not in RyR3. The crystal structures show that the phosphorylation loops are largely unstructured in RyR1 and RyR2 but more rigid in RyR3, implicating some correlation between the flexibility of the loop and the substrate feasibility. The structure of the Repeat34 domain from the insect *P. xylostella* has also been determined (Xu and Yuchi, 2019). Interestingly, the phosphorylation pattern of insect Repeat34 is temperature-dependent, which might be related to their environmental adaptation. Insect Repeat34 was found to possess several distinct features, such as an extra α-helix after the phosphorylation loop.

Repeat12 is located between SPRY1 and SPRY2 domains near the N-terminal region of the RyR sequence (Figure 2A). Although Repeat12 and Repeat34 have some structural similarities, the former is less symmetrical. This is mainly owing to the existence of an extra three-stranded β-sheet that fills a cavity between the two repeats of Repeat12 (Yuchi et al., 2015). The two repeats of Repeat12 are separated by a highly structured 30-residue loop, the equivalent of the phosphorylation loop in Repeat34, generating a U-shaped lid (“U-lid”) and interacting closely with an α-helix in Repeat12 (Figure 4D). According to the result of the docking experiment using the RyR1 cryo-EM maps, Repeat12 is located at the corners of the cytoplasmic domain, precisely above the SPRY2 domain. This location is corroborated by the high-resolution structures (Efremov et al., 2015; Yan et al., 2015; Yuchi et al., 2015). Because this corner region has been shown to be involved in the coupled gating of RyRs (Asghari et al., 2020), Repeat12 might play a role in this process.

### 2.2.3 SPRY

RyRs have three SPRY domains (SPRY1-3), which share high sequence identity between vertebrates and invertebrates. SPRY domains, located after the NTD of RyRs in sequence (Figure 2A), are named after two protein families, SPlA kinases and RYanodine receptors, where they were first identified. They have been implicated in protein-protein interactions in several protein families.

The crystal structures of the SPRY2 domains of RyR1 (residue 1,070–1,246 rabbit numbering) and RyR2 (residue 1,080–1,253 mouse numbering) isoforms have been determined with 1.34–1.84 Å resolution (Lau and Van Petegem, 2014). The structures reveal that two antiparallel β-sheets form a core, on which an N-terminal extension and a lid module further stabilize the folding of the domain (Lau and Van Petegem, 2014). The docking results reveal that the SPRY2 domain is located at a position connecting the NTD gating ring with the clamp region (Figure 2C).

The crystal structure of SPRY1 domain of RyR2 (residue 650–844 mouse numbering) was solved later at a resolution of 1.2 Å (Yuchi et al., 2015). SPRY1 shares similar core and lid modules with SPRY2, but it has a unique inserted “finger” module formed by a β-hairpin structure (Figure 4E). This “finger” protrudes out from the core and is stabilized by a conserved W713 residue at the base, which serves as an anchor point. The conformation of this finger is similar in both molecules of the asymmetric unit, and its residues are highly conserved among all three RyR isoforms, indicating that it has a key functional role as an anchor point for other RyR domains or auxiliary proteins. In addition, SPRY1 domain does not contain an “insertion loop,” which breaks a β-strand in two halves in SPRY2 (Lau and Van Petegem, 2014). Overall, despite having a similar core structure, SPRY1 and SPRY2 have diverged significantly through numerous insertions that alter their overall shape.

Although three SPRY domains were predicted to fold independently, the cryo-EM structures showed that they are partially intertwined. In RyR1, SPRY1 comprises two pairs of anti-parallel β-sheets (residues 1,466–1,491 and 1,615–1,634) from SPRY3 in addition to its own core β-sheets (residues 639–826). Similarly, SPRY2 contains a pair of anti-parallel β-sheet (residues 827–845) from SPRY1 (Yan et al., 2015). SPRY3 has a special structure consisting of two large structurally linked regions (residues 1,242–1,465 and 1,492–1,614) that are separated by an extension from SPRY1. A similar pattern is also observed in the RyR2 structure (Peng et al., 2016). Because of this domain organization, the crystal structures of individual SPRY1 and SPRY2 domains produced from recombinant expression cannot represent the whole domains, but rather the largest continuous core-forming segments (Lau and Van Petegem, 2014; Yuchi et al., 2015).

## 3 Structures of RyR Reveal the Disease Mechanisms

### 3.1 Crystal Structures of RyR Domains Containing Disease Mutations

More than 700 disease-associated mutations have been associated with RyRs, many of which are located in some mutation hotspot domains. X-ray crystallography has its advantage in studying the subtle structural changes caused by disease-causing mutations due to the high-resolution information. So far, the crystal structures of several mutant RyR domains, including NTD (Lobo and Van Petegem, 2009; Tung et al., 2010; Kimlicka et al., 2013a; Amador et al., 2013; Kimlicka et al., 2013b), SPRY1 (Touat-Hamici et al., 2021), SPRY2 (Lau and Van Petegem, 2014; Alvarado et al., 2019), Repeat12 (Yuchi et al., 2015), and Repeat34 (Yuchi et al., 2012), have been solved and provided insights into the mechanisms of these disease-causing mutations (Table 1).

### 3.1.1 NTD Mutant

The NTD is known as one of the three so-called “mutation hotspot regions,” containing fifty six mutations (http://www.hgmd.cf.ac.uk/ac/gene.php?gene=RYR) in both RyR1 (Amburgey et al., 2011; Snoeck et al., 2015) and RyR2 (Marjamaa et al., 2009). The structures of both mutant NTDs and their subdomains were studied thoroughly to acquire a better understanding of how a given mutation impacts the tertiary structure and consequently the gating of the channel. The NTD mutations are found to cluster in several main regions of both isoforms, including the central helix, various interfaces within and across subunits, and a hotspot (HS) loop (loop β8-β9 of NTD-A) (Amador et al., 2009; Tung et al., 2010; Kimlicka et al., 2013a; Kimlicka et al., 2013b; Borko et al., 2014). To date, fourteen crystal structures of mutant NTD have been solved, including L14R, G249R, C36R, V219I, I404M, R45C, D61N, and R402G mutants of RyR1 (Figure 4A) (Kimlicka et al., 2013a) and the A77V, V186M, R420Q, P164S, R169Q, and R176Q mutants of RyR2 (Figure 4B) (Lobo and Van Petegem, 2009; Amador et al., 2013; Kimlicka et al., 2013b; Borko et al., 2014).

The RyR1 mutant NTD structures show that most disease-causing mutations affect either the intra-subunit or inter-subunit domain-domain interface and alter relative domain orientations (Kimlicka et al., 2013a). Specifically, R45C, D61N, and R402G target ionic pairs between NTD-A and NTD-C: R45C abolishes a salt bridge with D447 on NTD-C, while D61N and R402G completely disrupt the ionic pair network. In general, they cause a large structural changes by affecting inter-domain ionic pairs. In contrast, a small number of mutations buried within the NTD domains, such as G249R and L14R, indirectly affect the inter-subunit interface through inducing conformational changes of residues at the interfaces. These findings highlight the overall importance of these interfaces in channel opening. However, some other mutations, such as V219I, I404M, and C36R, do not appear to induce any significant structural changes, but rather affect the thermal stability of the NTD protein. The C36R mutation showed the largest impact, lowering the melting temperature of RyR1 NTD protein by more than 9°C (Kimlicka et al., 2013a).

Several RyR2 NTD crystal structures have also been determined at atomic resolutions (Figure 4B). The structures of A77V (2.2 Å) and V186M (1.7 Å), which were solved on the background of RyR2 NTD-A, reveal that the mutations cause distinct local changes in the protein surface (Lobo and Van Petegem, 2009), while the structure of R420Q, which was solved based on RyR2 full NTD, shows that this CPVT mutation abolishes the chloride ion binding and reorients the three NTD subdomains (Kimlicka et al., 2013b). Other mutations, such as L62F, F329L, T415R, and L433P, located at the intra-subunit domain interfaces or buried inside the specific domains, induced protein instability, resulting in extensively degraded products (Kimlicka et al., 2013b).

In general, three molecular mechanisms are proposed for NTD mutations: 1. causing the misfolding of the protein; 2. destabilizing the interactions between NTD-A, NTD-B, and NTD-C; 3. affecting the interfaces between NTD and other RyR domains.

### 3.1.2 Repeat12 and Repeat34 Mutants

Several disease mutations have been identified in Repeat12 and Repeat34 domains, which were mapped to the high-resolution crystal structures of these domains (Yuchi et al., 2012; Yuchi et al., 2015) and also some of the mutant structures were determined by x-ray crystallography. Seven mutations were identified in the Repeat12 domain (Figure 4D), among which five of them from RyR1 (G893S, N899K, A933T, R1043G, and G1049S) are associated with MH (Levano et al., 2009), while the other two from RyR2 (R1013Q and R1051P) are associated with CPVT (Medeiros-Domingo et al., 2009). Several of these mutations are distributed on the surface, which are unlikely to cause misfolding of the domain but rather affect some domain-domain interactions in the full-length RyR. One exception is RyR1 R1043C, which affects a hydrogen bond network that is important for the stabilization of U-lid motif and causes a clear destabilization effect (Yuchi et al., 2015). RyR2 R1051P is located in the middle of the second α-helix of Repeat12. Thus, the substitution of the arginine with a proline, a “helix-breaking” residue, would probably disrupt this helix and possibly the structure of at least part of the domain.

Eleven disease mutations, including E2764K, S2776F, S2776M, L2785V, T2787S, R2840W, S2843P, L2867G, E2880K, R2939S, and R2939K, have been found in the RyR1 Repeat34 domain and associated with MH and CCD (Figure 4C) (Yuchi et al., 2012). Their distribution is not random but rather clustering into three structural regions. One mutation, L2867G, which targets a buried hydrophobic residue, induces a significant thermal instability and aggregation at room temperature. All of other mutations are exposed to the surface. Seven of them cluster on the same side as the phosphorylation loop. Mutations in the phosphorylation loop can either directly abolish the S2843 phosphorylation (S2843P) site directly or remove a positive charge (R2840W). Removing a positive charge has the same effect as the adding a negative charge *via* phosphorylation. The proximity of the majority of the mutations in Repeat34 to the phosphorylation sites implies that the mutations may influence the same interaction with a neighboring domain or a regulatory subunit as phosphorylation does. The remaining three mutations, R2939S, R2939K, and E2880K, are located on the completely opposite side of the domain. Crystal structures reveal that R2939S and R2939K can affect intra-domain salt bridges and hydrogen bonds with E2870 and Q2877, while E2880K simply affects the local surface charge properties.

Four mutations (D708N, N759D, R739H, and I784F) identified in SPRY domains have been associated to myopathies (Figure 4E). D708N in RyR1 has been linked to MmD and atypical periodic paralysis (Zhou et al., 2010). Based on the crystal structure, the equivalent residue in RyR2, D720, forms a salt bridge with the equally conserved R694. D720 is part of the “finger” motif, mediating the SPRY1-SPRY2 interaction. Thus, a mutation to asparagine would weaken the interaction and consequently disrupt the SPRY1-SPRY2 interaction (Yuchi et al., 2015). Furthermore, considering the direct involvement of SPRY1 in FKBP binding, the question emerges whether the SPRY1 disease mutations directly affect FKBP binding. The mutation of SPRY1 N760D mutation [equivalent to the human N759D core myopathy mutation (Bharucha-Goebel et al., 2013)] lies at the FKBP interface. Interestingly, this mutation leads to a four-fold decrease in total FKBP binding at saturating levels without affecting FKBP binding affinity (Yuchi et al., 2012). The mutation significantly reduced the expression level of SPRY1 N760D, implicating that the residue is critical in folding of the domain, but is less important in maintaining this structure as a receptor of FKBP. Two mutations, including R739H associated with CPVT (Medeiros-Domingo et al., 2009) and I784F associated with short-coupled torsade de pointes Touat-Hamici et al. (2021), were identified in RyR2 SPRY1. Recently, Touat-Hamici et al. solved a crystal structure of RyR2 SPRY1 I784F at 1.21 Å. The structure revealed that I784F causes a conformational change in a loop at the interface with SPRY3 and Repeat12, which affects inter-domain interactions and alters channel gating. In addition, this mutation has shown to reduce the melting temperature of SPRY1 by 7°. The impacts in both structure and thermal stability might contribute to the increase of propensity for spontaneous Ca^2+^ release observed in the functional assays.

Five mutations, including R1075W, G1165D, R1179W, R1127H, and R1140C, have been identified in the SPRY2 domain (Lau and Van Petegem, 2014). G1165D and R1075W affect partially buried residues and were associated to CCD (Böhm et al., 2013). Despite both mutations were found in conjunction with other mutations elsewhere in RyR1, they interfere with proper folding and should either contribute or be entirely responsible for the disease phenotype (Lau and Van Petegem, 2014). For the three mutations at the domain surface, R1179W, R1127H, and R1140C, they likely lie at some interfaces with other RyR domains or an auxiliary protein to become disease causing. Generally, the mutations that interfere with the folding of SPRY2 result in loss-of-function phenotypes, whereas mutations on the surface result in gain-of-function ones. In RyR2 SPRY2, T1107M has been associated with hypertrophic cardiomyopathy as well as CPVT (Figure 4F) (Medeiros-Domingo et al., 2009). The functional experiments have convincingly demonstrated that it confers an uncommon loss-of-function phenotype with early termination of Ca^2+^ release (Tang et al., 2012). The crystal structure of RyR2 SPRY2 A1107M (equivalent mutation in mouse RyR2) shows that the mutation abolishes a surface salt bridge between two neighboring β-strands to accommodate this nearby bulky methionine residue, explaining the observed significant thermal destabilization, resulting in 22% unfolding at physiological temperatures and the loss-of-function phenotype (Lau and Van Petegem, 2014). Recently, another RyR2 mutation discovered in a patient from a genotype-negative Hypertrophic cardiomyopathy (HCM) cohort, P1124L, was structurally and functionally characterized (Alvarado et al., 2019). HEK293 cells expressing recombinant RyR2 P1124L displayed a cytosolic loss-of-function phenotype and a higher sensitivity to luminal Ca^2+^. This mutation induces significant conformational changes in SPRY2, which might disrupt a nearby interface between SPRY2 and SPRY3 and alter the channel gating property.

### 3.2 Cryo-EM Structures of RyR Containing Disease-Associated Mutations

#### 3.2.1 The Distribution of Disease-Causing Mutations in RyRs

**FIGURE 5:**
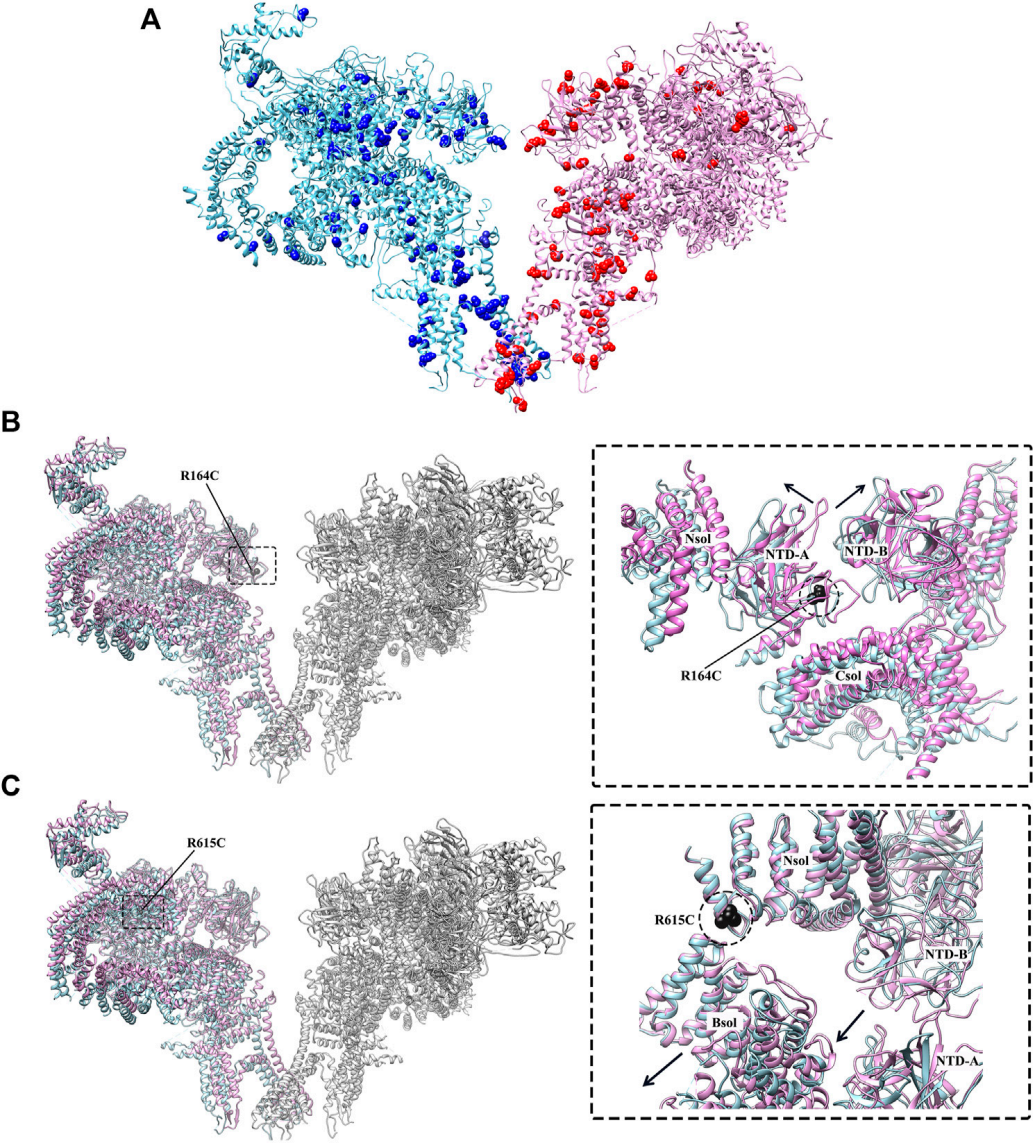
Disease-causing mutations of RyR studied by cryo-EM. **(A)** The distribution of disease-causing mutations (spheres) in RyR1 (light blue) and RyR2 (magenta). One protomer is shown for each isoform. **(B)** Superposition of the structures of WT RyR1 (magenta) (PDB: 3J8H) and RyR1 R164C (light blue) (PDB: 6WOT). **(C)** Superposition of the structures of WT RyR1 (magenta) (PDB: 6W1N) and RyR1 R615C (light blue) (PDB: 6X34). Left panels of. **(B,C)** show the locations of the mutations in the full-length RyR1, and the right panels show the relative displacement induced by the disease-causing mutations in zoomed-in views.

To date, over 700 mutations have been identified in both RyR1 and RyR2, and these are distributed throughout the receptor (Figure 5A). Several attempts have been sought to establish a relationship between the 3D structural distribution of these disease-associated mutations and their disease phenotype and severeness with the aim to create some prognostic markers. Roston et al. created a 3D model based on the available crystal structures of RyR2 domains and cryo-EM structures of rabbit RyR1, which can be used to predict the structural and functional impacts of CPVT-related variants. Interestingly, they discovered that the majority of the CPVT mutations cluster along the four-fold symmetry axis with almost none in the peripheral region. The mutations with severe clinical phenotype, namely cardiac arrest, are distributed in a few important interfaces in RyR2 structure, including NTD, S4-S5 linker, and the pore-forming domain (Roston et al., 2018). NTD forms a continuous gating-ring structure surrounding the four-fold symmetry axis and is vital in regulating channel gating. The S4-S5 linker and the pore-forming domain belong to the transmembrane part of the channel that directly mediates calcium permeation. The linker between S4 and S5 has been demonstrated to be a key allosteric coupling element between the signal-sensing modules and the channel gates in several ion channel families (Blunck and Batulan, 2012), while the pore-forming domain, comprised of helices S5 and S6, creates the minimal “channel” through which calcium ions can permeate the SR membrane. In contrast, the mutations with relatively mild phenotype, such as syncope, are scattered in other interfaces. The distribution pattern of mutations in the 3D structure of RyR is useful in the prediction of the severeness of newly identified mutations during diagnosis in the future.

#### 3.2.2 The Impacts of Disease-Causing Mutations Revealed by Cryo-EM Structures

Although the crystal structures of mutant RyR domains provide valuable information about their localization and impacts on the local structural changes, they were not able to reveal the global and long-range allosteric structural changes of the channel caused by these mutations. Recently, several disease-causing mutations of RyR1 were investigated through cryo-EM (Iyer et al., 2020; Woll et al., 2021), provided insights into their disease mechanisms. Iyer et al. solved the cryo-EM structures of two gain-of-function RyR mutations, RyR1 R164C and RyR2 R176Q, both located in an equivalent position of NTD-A, that are associated with MH and CPVT, respectively (Iyer et al., 2020). Despite a comparable loss of positive charge, the structures revealed that the mutations had an isoform-specific effect on the structure of RyR. A salt bridge network is disrupted by RyR1 R164C mutation, resulting in a 1 Å shift and 6° rocking of the NTD-A, which induces the channel into an altered pre-activated conformation (Figure 5B). This movement is similar to the movements in these domains that occur upon channel opening, but lesser in magnitude (6.5 Å shift and 8° rocking for channel opening). However, there was no significant change of the NTD-A^+^/NTD-B distance for RyR2 R176Q, and the NTD-A did not shift in orientation.

In another recent study, Woll et al. solved the cryo-EM structure of a pig RyR1 containing an MH-associated mutation, R615C. The structure reveals that the mutation in the N-solenoid (Nsol) domain interacts with N1678 in junctional solenoid (Jsol) and E2175 in bridging solenoid (Bsol) (Figure 5C) (Woll et al., 2021). Previous cryo-EM studies revealed that the disruption of the interaction between Nsol and Bsol needs to occur upon channel opening. R615C mutation affects an interface between three solenoid domains and facilitates channel opening by causing a 2–3 Å shift in the Bsol near E2175 and a secondary movement of ∼10 Å in the Bsol around residue 2,457 to reach an “intermediate” state (Figure 3C). Furthermore, Woll et al. also showed that apo-CaM binding abolishes the “intermediate” state caused by the mutation and induce the channel into the open state. In the WT channel, the apo-CaM N-lobe contacts the Bsol (residues 2,190–2,242, interface 1) and is close to a short loop in the Bsol (residues 2,595–2,600, interface 2). The apo-CaM C-lobe interacts with Bsol (residues 3,627–3,634, interface 3) immediately downstream of the Jsol (residues 1,975–1,999, interface 4). These interactions induce a conformational change in Jsol, bringing interface 4 closer to the apo-CaM C-lobe while having no discernible effect on the structure of the closed channel pore. In contrast, apo-CaM binding to R615C channels alters the tilt angle between the Nsol and Bsol, causing it to more similar to this region of the open channel. The R615C-induced change in the Nsol-Bsol interface reduces the energy barrier for channel opening, contributing to the increased sensitivity of the mutant channel to open in response to activators. The cryo-EM classification results showed that WT pRyR1 in the presence of apo-CaM and in the absence of ATP produced mostly closed channels (27% open), whereas under the same condition the R615C mutation increase the open state population by ∼2.5-fold (69% open), suggesting that the mutation increases the opening probability of the channel (Woll et al., 2021). Overall, this finding highlights the role of the solenoid regions and CaM as key elements in propagating the effects of a disease-causing mutation. A common theme of gain-of-function RyR mutations revealed by Iyer et al. (2020) and Woll et al. (2021) is that the MH mutations can lead to some distinct “intermediate” pathological local conformations and cause the cytoplasmic regions of RyR1 to more closely resemble those of the open channel, thereby facilitating channel opening.

More mutations have been studied by molecular dynamic (MD) simulations based on the structural templates provided by cryo-EM and X-ray crystallography. MD simulations can provide information about dynamics that are difficult to capture by cryo-EM and X-ray crystallography. Zheng and Liu. (2017) studied the structural impacts of three disease mutations, K155E, R157Q, and R164Q (corresponding to mutations K167E, R169Q, and R176Q in RyR2), on the structure of RyR1 NTD tetramer. They revealed a dynamic network of inter-subunit salt bridges that critically control the relative motions and stability of the NTDs in RyR1, and are disrupted by the above mutations. Another study by Xiong et al. (2018). showed a CPVT mutation, A165D, located at the same inter-subunit interface of NTD can also perturb the conformation of the closed-state tetramer structure of NTD, suggesting that a similar disease mechanism of NTD mutations applies to both RyR1 and RyR2 given the strong conservation of NTD between them. Overall, structural analysis suggests that A165D, K167E, R169Q, and R176Q mutations all cause structural disruptions in a similar manner.

Different disease-causing mutations, depending on their locations in the structure of RyR, are likely to have different impacts on the local and global structures. Three mutation clusters, or so-called “hotspots,” have been previously identified in the RyR, including the NTD (35–614, human RyR1 numbering), the central domain (2,129–2,458), and the CTD (3,916–4,942) (Betzenhauser and Marks, 2010), which implies a functional linkage between these domains. In the “zipping/unzipping” hypothesis raised in early 2000s, the mutations were proposed to be located in the interdomain interfaces and destabilize the closed state of the channel by impairing crucial interdomain interactions (Ikemoto and Yamamoto, 2002; Uchinoumi et al., 2010). As a result, these mutations cause an aberrant sensitivity of the channel to activating signals, including caffeine, halothane, and 4-chloro-m-cresol (Tong et al., 1999; Balog et al., 2001), or displaces the inhibitory proteins, such as CaM, to cause CPVT arrhythmogenesis (Nakamura et al., 2019). The “zipping/unzipping” hypothesis provides a reasonable explanation of disease mechanism. The high-resolution cryo-EM structures of RyR in the different functional and pathological states provided valuable information about the gating and disease mechanisms, but the limited numbers of snapshots are still not enough to provide high resolution at the time-scale and describe the detailed conformational changes related to long-range allosteric domain linkages and the regulation of this large protein with a variety of modifying factors. On one hand, more cryo-EM structures describing the impacts of mutations from different interfaces will give a more comprehensive view of the pathological mechanism. On the other hand, the time-resolved cryo-EM or long MD runs combined with cryo-EM structures might provide further insights into the “functional linkage” by revealing the dynamic changes.

## 4 Endogenous and Exogenous Modulator Regulating Ca^2+^ Release in RyR

**FIGURE 6:**
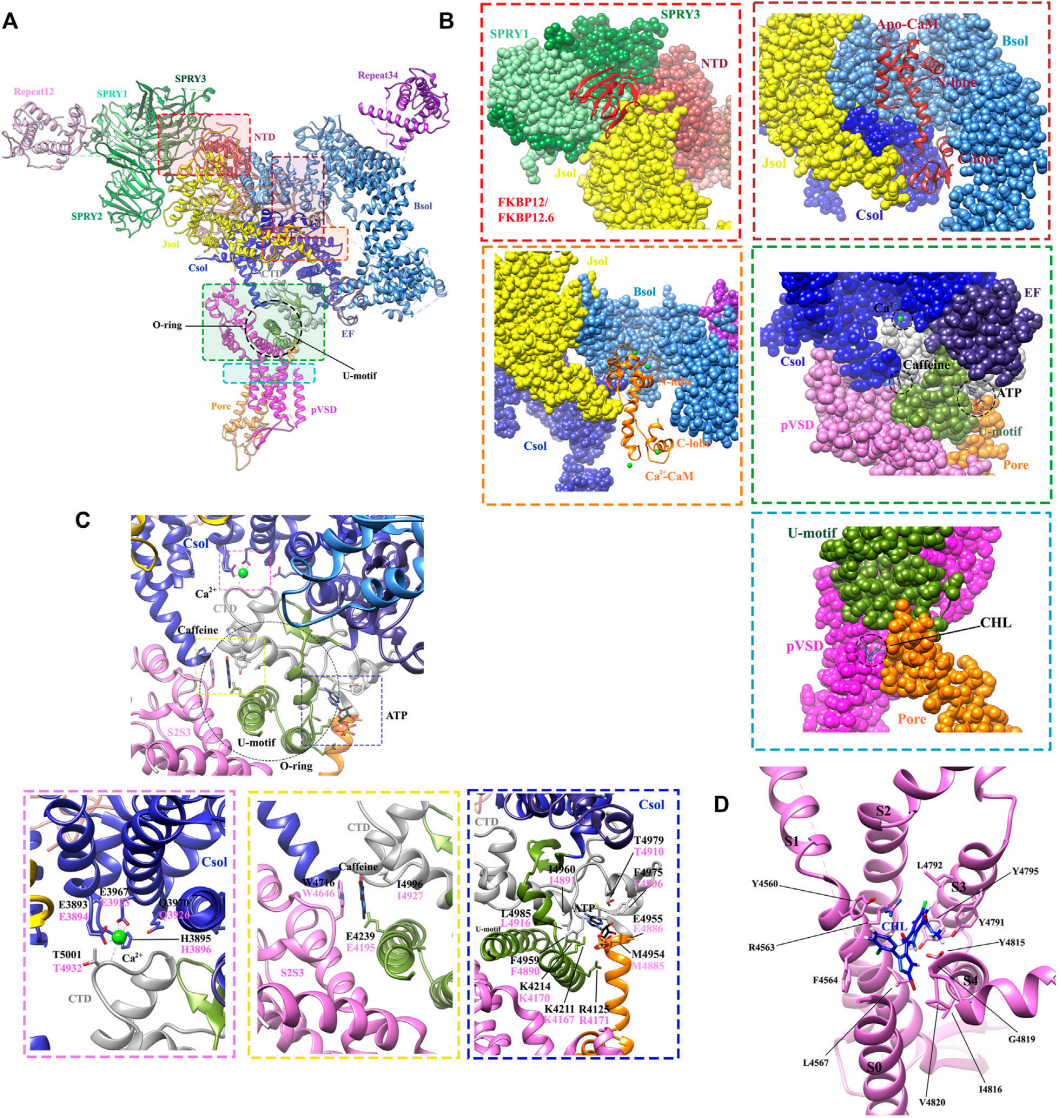
Structures revealing the modulations of RyRs by the endogenous and exogenous modulators. **(A)** The locations of different ligand-binding sites within the full-length RyR1 (PDB: 6M2W) are indicated by colored boxes. A single protomer is shown. **(B)** The zoomed-in views of the binding sites for FKBPs (red dot square), apo-CaM (brown dot square), Ca^2+^-CaM (orange dot square), Ca^2+^, ATP, caffeine (green dot square), and chlorantraniliprole (CHL) (blue dot square). The different domains are displayed in surface view by the same color scheme as. **(A,C)** Zoomed-in view of the binding sites for Ca^2+^ (magenta dot square), caffeine (yellow dot square), and ATP (blue dot square) in RyR1. The contact residues are labelled in black (RyR1) and magenta (RyR2). **(D)** Zoomed-in view of the CHL binding site in RyR1. The detailed interactions and ligand-coordinating residues are labeled in. **(C,D)**.

RyR is one of the biggest known ion channels. It is activated predominantly by Ca^2+^ but also regulated by many factors, including Mg^2+^, ATP, phosphorylation, the redox potential, and via the interactions with several modulatory proteins, such as DHPR, FKBP, CaM, etc. The regulations of RyR by several proteins [calsequestrin (Beard and Dulhunty, 2015), sorcin (Meyers et al., 1995), triadin (Chopra et al., 2009), homer (Pouliquin and Dulhunty, 2009), histidine-rich Ca^2+^ binding protein (Arvanitis et al., 2018), S100A1 (Rebbeck et al., 2016), and junctin (Li et al., 2015)] and pharmacological agent [(Paul-Pletzer et al., 2002; Kobayashi et al., 2005; Oo et al., 2015; Diszházi et al., 2019), flecainide (Hilliard et al., 2010), K201 (Kaneko et al., 2009), S107 (Bellinger et al., 2008; Lehnart et al., 2008; Bellinger et al., 2009), scorpion toxins imperatoxin A (Gurrola et al., 2010), maurocalcine (Esteve et al., 2003), hemicalcin (Shahbazzadeh et al., 2007), hadrucalcin (Schwartz et al., 2009), ryanodine (Meissner, 1986), diamide (Ma et al., 2020), and carvedilol (Zhou et al., 2011)] have been extensively characterized by electrophysiological and biochemical experiments (Santulli et al., 2018), and reviewed elsewhere (Lanner et al., 2010; Van Petegem, 2012; Meissner, 2017; Woll and Van Petegem, 2021). Recently, several high-resolution structures of RyRs in complex with different modulators have been determined (Wu et al., 2015; Bai et al., 2016; des Georges et al., 2016; Wu et al., 2016; Chi et al., 2019; Gong et al., 2019; Ma et al., 2020), revealing a wealth of information about the ligand-binding sites, the detailed interaction modes, and the conformational changes induced by these molecules. In this section, we mainly focus on the recent structural studies of some important RyR modulators (Figures 6A,B), including both endogenous and exogenous ones.

### 4.1 Endogenous Modulators

#### 4.1.1 Calcium Ion

The determination of the high-resolution cryo-EM structures of RyR1 and RyR2 isoforms has shed light on the intricate regulatory mechanism governing Ca^2+^ modulation (des Georges et al., 2016; Chi et al., 2019). Several studies previously reported that RyR channel contains a moderate-affinity micromolar Ca^2+^-binding site for Ca^2+^-dependent activation and a lower-affinity millimolar Ca^2+^-binding site for Ca^2+^-dependent inhibition (Watras and Ehrlich, 1991; Clapham, 1995). The cryo-EM structures of rabbit RyR1 solved by des Georges et al. showed that the moderate-affinity of Ca^2+^-binding site at the interdomain interfaces between Csol and CTD is highly occupied at 30 μM Ca^2+^ concentration (Figure 4A), and the Ca^2+^-coordinating residues (E3893, E3967, T5001, Q3970, and H3895) are conserved between RyRs and IP3Rs (des Georges et al., 2016). They propose that the binding of 30 μM Ca^2+^ primes the channel and makes it ready for activation, and the binding of other channel activators, e.g., ATP and caffeine, would induce the channel to a fully open-state (Figure 6C) (des Georges et al., 2016). This observation was supported by the reports from other independent structural studies on RyR1 (Efremov et al., 2015; Ma et al., 2020). Later, Yan’s group investigated whether Ca^2+^ alone could open RyR2 in the absence of other modulators using cryo-EM. They discovered that the channel is inactivate in the absence of Ca^2+^, but that increasing Ca^2+^ to 20 μM concentration with the addition of PCB95 in the absence of FKBP12.6 (Peng et al., 2016) or with the simultaneous addition of ATP and caffeine in the presence of FKBP12.6 (Gong et al., 2019) induces a contraction of the central domain, which applies a pulling force, facilitating the dilation of the S6 bundle and opens the pore. Overall, these studies reveal the location of the moderate-affinity Ca^2+^-activation site and indicate that Ca^2+^ is essential for the opening of RyRs, and multiple modulators highly regulate the calcium-induced channel opening by stabilizing the open-state of the channel. In contrast, the location of the low-affinity Ca^2+^-binding site remains elusive probably due to the low occupancy at the experimental condition, although a study using the chimeric channels between RyR1 and RyR2 suggests that it might be located in one of the two EF hands (Gomez and Yamaguchi, 2014).

#### 4.1.2 ATP and Magnesium Ion

Adenine-derived nucleosides and nucleotides are competitive partial agonists of both RyR1 (Laver et al., 2001) and RyR2 (Kermode et al., 1998). ATP enhances the maximal opening probability (P_O_) of the channel, without affecting its conductance (Kermode et al., 1998). des Georges et al. (2016). solved cryo-EM structure of ATP-binding site in RyR1 in the absence or presence of other modulators, revealing that ATP-binding site is constituted by the cytoplasmic extension of S6 and C-terminal components. M4954, F4959, T4979, and L4985 intimately interact with adenine-base; three positive residues, K4211, K4214, and R4215 contact with the negatively charged triphosphate tail; while E4955 appears to contact with the ribose ring. Similar to the structure of RyR in Ca^2+^ alone condition, ATP alone also primes the channel and the addition of other activators are required to open RyR1, especially Ca^2+^ (Figure 6C).

Mg^2+^ was proposed to have a complex role on RyR activity: it acts as a competitive inhibitor by binding to the moderate-affinity Ca^2+^ site which is essential for Ca^2+^-dependent activation, but also shows inhibitory effects on RyRs by binding to the low-affinity inactivation site with similar affinity as Ca^2+^ (Laver, 2018). In addition, Mg^2+^ also binds to the luminal regulatory site and reduces the conductance of Ca^2+^ through the channel (Gusev and Niggli, 2008; Laver and Honen, 2008).

In addition, the majority of cellular ATP appears as MgATP complex, which is the most physiologically active form of ATP in cells. An increase in Mg^2+^ concentration, which increases MgATP and decreases non-complexed ATP, also decreases RyR activity (Walweel et al., 2014). However, whether MgATP or ATP is a physiological regulator of RyRs remains debatable. It is argued that RyR can be activated by “free” ATP because ATP activates RyRs in the absence of Mg^2+^ and because three positively charged amino acid residues in the ATP-binding site balance out the negatively charged triphosphate tail of ATP (des Georges et al., 2016). Experimental evidence on the preference of ATP or MgATP in physiological regulation of RyR has not yet been provided (des Georges et al., 2016)

#### 4.1.3 Calmodulin

CaM binds to RyR under both apo-CaM and Ca^2+^-CaM conditions and exhibits biphasic regulation of RyR1. Briefly, it inhibits both RyR1 and Gomez and Yamaguchi, 2014RyR2 at high Ca^2+^ concentrations, while activating RyR1 but inhibiting RyR2 at low Ca^2+^ concentrations (Fruen et al., 2000; Balshaw et al., 2001). As a result, CaM regulates RyRs in an isoform-specific manner. The binding of CaM has been widely investigated *via* X-ray crystallography and cryo-EM, demonstrating that both apo-CaM and Ca^2+^-CaM may bind to the periphery of the cytosolic cap, although the exact binding mode relies on the Ca^2+^ concentrations (Samsó and Wagenknecht, 2002; Huang et al., 2012).

The crystal structures of Ca^2+^-CaM bound to the calmodulin-binding domain (CaMBD2) of RyR1 and RyR2 have been solved (Maximciuc et al., 2006; Holt et al., 2020). The binding modes of both isoforms are similar, with W3620 (RyR1 numbering) interacting with a hydrophobic pocket of the C-lobe and F3636 (RyR1 numbering) interacting with the N-lobe. This dual binding mode explains the nanomolar affinity of the CaMBD2 peptide for Ca^2+^-CaM.

The recent cryo-EM structures of RyRs in complex with CaM confirmed the findings from the crystal structures and shed light on the regulation of RyR channel gating by CaM (Figure 6B). Gong et al. (2019) solved the cryo-EM structures of RyR2 in complex with apo-CaM (resolution of 3.6 Å) and Ca^2+^-CaM (resolution of 4.4 Å). The structures show that apo-CaM and Ca^2+^-CaM establish different but overlapping contacts in an elongated cleft formed by the handle, helical, and central domains, which is consistent with prior low-resolution structures of RyR1 (Samsó and Wagenknecht, 2002; Huang et al., 2012). Upon binding of Ca^2+^-CaM, both N- and C-lobes of CaM wrap around an α-helix by interacting with the same two aromatic anchors (F3604 and W3588 in RyR2) in their hydrophobic cavities as revealed by the previous crystal structures (Figure 6B) (Maximciuc et al., 2006). In contrast, for apo-CaM, the N-lobe of CaM contacts with the region of BSol domain, whereas the C-lobe lies in the JSol domain, the binding and conformation of apo-CaM bound to the RyR2 are similar to the ones of CaM1234 bound to RyR1 (Ma et al., 2020). These structures were further validated *via* mutagenesis analysis (Gong et al., 2019). The binding of Ca^2+^-CaM stabilizes the RyR in a closed-state. Ca^2+^-PCB95, which normally facilitates channel opening, can be counteracted effectively by Ca^2+^-CaM, which keeps the channel closed. However, the RyR in complex with Ca^2+^-CaM remains open in the presence of caffeine and ATP. This suggests that CaM acts only as one modulator of the channel whose conformational and functional state is delicately regulated by the presence and absence of a combination of positive and negative regulators.

#### 4.1.4 FKBP12 and FKBP12.6

Two FKBPs, FKBP12 (also named calstabin1) and FKBP12.6 (also named calstabin2), are generally regarded to be components of the massive RyR complexes and selectively associated with RyR1 (FKBP12) and RyR2 (FKBP12.6) in an isoform and tissue specific manner (Chelu et al., 2004; Santulli and Marks, 2015). They stabilize the closed state of the channel and prevent intracellular Ca^2+^ leak (Ahern et al., 1997; MacMillan, 2013; Santulli and Marks, 2015). An early investigation demonstrated that a hydrophobic cluster within SPRY1 is required for FKBP binding based on the combined results from crystal structures, FRET experiment, mutagenesis, and molecular docking to cryo-EM maps (Yuchi et al., 2015). Later, low and high resolution of cryo-EM studies has determined the binding sites of FKBP12 and FKBP12.6 on RyR1 at the periphery of the tetrameric assembly (Samsó et al., 2006; Yan et al., 2015; Zalk et al., 2015). FKBP binds at the N-terminus of the BSol (Figure 6B). It stabilizes the link between the pore and cytoplasmic region by rigidifying the interface between BSol and SPRY1/SPRY2 (Yan et al., 2015; Zalk et al., 2015; des Georges et al., 2016).

The regulation of RyR2 by FKBP is complicated and controversial. While FKBP12.6 has a higher affinity to RyR2, FKBP12 is more abundant in the heart (Galfre et al., 2012). Some studies showed that FKBP12 acts as a high-affinity activator of RyR2 to sensitize the channel to cytosolic Ca^2+^, while FKBP12.6 has lower efficacy but can antagonize the effect of FKBP12 (Galfre et al., 2012). But other studies support that FKBP12.6-binding promotes the closed state of RyR2 and inhibits its function (Marx et al., 2000; Wehrens et al., 2003). This avenue of thought is corroborated by a number of studies showing that lack of binding of FKBPs causes leaky RyRs in pathologies such as muscular dystrophy (Fauconnier et al., 2010), sarcopenia (Andersson et al., 2011), cardiac arrhythmias (Wehrens et al., 2004a), and heart failure (Marx et al., 2000). In this scenario, overexpression of FKBP12.6 likely to serve as an inhibitor of arrhythmogenesis by lowering diastolic RyR2 Ca^2+^ leak and halting spontaneous Ca^2+^ release (Gómez et al., 2004; Guo et al., 2010; Zhang et al., 2016). Fernández-Morales et al. (2022) reported that RyR2 N771D mutation located in FKBP-binding site (corresponding to N760D in RyR1) has a minor effect on Ca^2+^ signaling but promotes “arrhythmogenesis” in human stem cells derived cardiomyocytes. In a recent study Chi et al. (2019), solved the cryo-EM structures of RyR2 in the absence or presence of FKBP12.6 at a resolution of 6.1 and 4.6 Å, respectively. FKBP12.6 causes relaxation in the central domain of the channel, stabilizing RyR2 in a closed state in the presence of Ca^2+^-PCB95, Ca^2+^-ATP, or Ca^2+^-caffeine, indicating that FKBP12.6 is involved in pathophysiological regulation of RyR2 (Marx et al., 2000; Wehrens et al., 2003). In the presence of FKBP12.6, however, combining the synergistic effects of Ca^2+^-ATP or Ca^2+^-caffeine is sufficient to open the channel (Chi et al., 2019).

#### 4.1.5 Ca_v_1.1, STAC3, and Junctophilin

The voltage dependent Ca^2+^ channels (Ca_V_1.1 and Ca_V_1.2) are also referred as dihydropyridine receptors (DHPRs). Ca_V_1.1 is expressed in skeletal muscle, whereas cardiac myocytes express Ca_V_1.2 with a trace of Ca_V_1.3 in atrial myocytes (Zamponi et al., 2015). Ca_V_1.1 modulates RyR1 activity through direct mechanical interactions, while Ca_V_1.2 modulates RyR2 by an indirect Ca^2+^-induced Ca^2+^ release (CICR) release pathway. RyR1 is activated physiologically *via* direct physical interaction with the Ca_V_1.1 complex and the surrounding RyR1 tetramers in the crystal line-like assembly (Tanabe et al., 1990; Protasi et al., 1997). Several domains of the cytoplasmic region of RyR1, such as SPRY3 domain, were shown to be involved in coupling with the Ca_V_1.1 complex (Perez et al., 2003). The architecture organization of a pseudotetrameric eukaryotic Ca_V_ channel in complex with its auxiliary subunits has been solved (Wu et al., 2015; Wu et al., 2016), advancing our understanding of EC-coupling mechanism and providing a three-dimensional template for molecular interpretations of Ca_V_ and Na_V_ channel functions and disease mechanisms. Although, the structures of Ca_V_1.1 (Wu et al., 2015; Wu et al., 2016; Zhao et al., 2019) and RyRs (Efremov et al., 2015; Yan et al., 2015; Zalk et al., 2015; Bai et al., 2016; Peng et al., 2016; Wei et al., 2016; Chi et al., 2019; Gong et al., 2019; Ma et al., 2020) have been solved individually, the detailed interaction mode between these two channels remains elusive. Furthermore, the key elements in Ca_V_ for RyR1 binding, such as the II-III loop of the α1 subunit and part of the β subunit, are still missing from the determined Ca_V_ structure. Bai et al. (2016). docked structures of the Ca_V_1.1 complex (PDB: 3JBR) and RyR1, hypothesizing that domains of Cav1.1α1 would cause shifts of the β-subunit and other cytoplasmic segments of Ca_V_1.1 upon depolarization, which might prompt movement of the neighboring cytoplasmic RyR1, such as SPRY3 domain. Perni et al. (2017) showed that the functional coupling can be reconstituted in tsA201 cells by expressing five junctional proteins, Ca_V_1.1, RyR1, β1a, STAC3, and junctophilin2, highlighting the importance of these proteins in skeletal muscle EC-coupling. Ultimately, the determination of a cryo-EM structure of EC-coupling super-complex would provide a long-awaited answer to how this complex molecular machine works.

STAC3 is an essential protein for EC-coupling and acts as the auxiliary protein linking Ca_V_1.1 and RyR1. The interactions between STAC3 and Ca_V_1.1 are well-known and functionally validated (Polster et al., 2018; Rufenach et al., 2020). The crystal structures of tandem-SH3 domains of different STAC isoforms was solved up to 1.2 Å resolution. Combined with the results from ITC and calcium imaging experiments, they proved that STAC3 binds to Ca_V_1.1 through interacting with the II-III loop of the α1 subunit of Ca_V_1.1 (Yuen et al., 2017).

Junctophilins (JPHs) are known to stabilize the structure of the junctional membrane complex by bridging the plasma membrane and the sarcoplasmic membrane. In muscle tissue, JPHs allow for the communication between Ca_V_, located in the transverse-tubule (T-tubule) membrane, and RyRs in the SR membrane. Currently, the structure of a JPH2 alone and in complex with Ca_V_1.1 has been solved (Yang et al., 2022), showing that this interaction is required for clustering of these channels and for robust muscle EC-coupling. However, the complex structure of JPHs-RyRs is still a missing jigsaw piece, with which one can appreciate the assembly of the full Ca_V_-JPH-RyR complex.

#### 4.1.6 PKA and CaMKII

The large cytoplasmic region of RyRs has numerous phosphorylation sites (Takeshima et al., 1989) that can be targeted by protein kinases, including cAMP-dependent protein kinase (PKA) and CaM-dependent kinase II (CaMKII) (Dulhunty et al., 2001; Marx et al., 2001). However, the location and physiological importance of many phosphorylation target sites remains questionable. Among, the best studied sites are RyR2 S2808 (S2843 in RyR1) targeted by PKA (Witcher et al., 1991; Marx et al., 2000) and S2814 targeted by CaMKII (Wehrens et al., 2004b). These two residues are found within the Repeat34 domain, in a linker loop (phosphorylation loop) connecting Repeats3 and 4 (Yuchi et al., 2012). Crystal structures of the Repeat34 domain of all three mammalian isoforms have been determined (Yuchi et al., 2012). The electron density of the phosphorylation loop is low in both RyR1 and RyR2 crystal structures, preventing direct visualization of the phosphorylation target sites. The flexibility of the phosphorylation loop might be essential for the recognition by the kinases because the linker in non-phosphorylatable RyR3 is more structured.

Recently, Haji-Ghassemi et al. (2019) solved a crystal structure of the mouse RyR2 Repeat34 domain bound to the catalytic domain of PKA (PKAc) in complex with an ATP analogue, revealing the detailed interactions between the phosphorylation loop and the active site of PKAc. The activity of PKA can be positively or negatively regulated by different phosphorylation patterns of this loop. In addition, they solved the structure of PKAc in complex with the CaMKII phosphomimetic mutant (S2814D). The mutation induced the formation of a new ɑ-helix which promotes the binding of PKAc to RyR2 Repeat34. This implies that there is substantial cross-talk between different kinase pathways: the phosphorylation by CaMKII at one site can enhance the phosphorylation level of another site by PKA.

In addition to phosphorylation, other types of post-translational modifications (PTM), such as oxidation, nitrosylation, and glutathiolation, can also modulate the open probability and gating behavior of RyRs (Sun et al., 2003; Denniss et al., 2018). RyR has 80–100 cysteines per monomer, of which about 25–50 cysteines are in the reduced state and 6–8 are regarded as “hyperreactive” (Xu et al., 1998; Dulhunty et al., 2000). The oxidation, nitrosylation or glutathiolation of critical sulfhydryls on the cytoplasmic region of RyR1 and RyR2 affects the gating properties and the sensitivity to modulators such as ATP, caffeine, Ca^2+^, Mg^2+^, calmodulin (Zhang et al., 1999), and FKBP (Aracena et al., 2005). Ca^2+^ efflux from the cardiac SR vesicles is increased by reactive oxygen, and calmodulin has been identified as a mediator of reactive oxygen-triggered Ca^2+^ release *via* the RyR (Kawakami and Okabe, 1998; Xu et al., 1998; Sun et al., 2003; Voss et al., 2004; Aracena-Parks et al., 2006; Gangopadhyay and Ikemoto, 2006; Gonzalez et al., 2010). The large number of PTMs and their heterogeneity make it difficult to obtain the high-resolution structures and study their specific impacts. The recent development of genetic code expansion technique could provide powerful tools to generate homogeneously modified proteins suitable for high-resolution structural studies.

### 4.2 Exogenous Modulators

#### 4.2.1 Ryanodine and Caffeine

Ryanodine and caffeine have been extensively used in controlling cytoplasmic and ER luminal Ca^2+^ concentrations. The ryanodine, a plant alkaloid, affects RyR function in two ways: at low nanomolar concentrations it locks the channel in a subconductance state, while at high micromolar concentrations it blocks the conductance (Meissner, 1986). According to the results of [^3^H]Ryanodine binding studies, binding to a single high-affinity site locks RyRs into an open subconductance state, while binding to one or more ryanodine to low-affinity sites totally abolishes the current (Lai et al., 1989; Pessah and Zimanyi, 1991). According to a cryo-EM structure (des Georges et al., 2016), the high-affinity ryanodine-binding site is located within the pore, close to Q4933, and ryanodine binding to this site causes the transmembrane pore to dilate. The density does not match the exact shape of ryanodine molecule. This is because only one molecule of ryanodine binds to the site in the pore. Due to the C4 symmetry applied during the data processing this ryanodine molecule was averaged over four symmetrically equivalent sites. However, the mutation in this site specifically reduces ryanodine binding without affecting channel function, proving this is the real high-affinity binding site of ryanodine (Fessenden et al., 2001).

The caffeine binds to a site located between the S2S3 domain and the CTD in RyR1 (Figure 6C). Caffeine is sandwiched by the hydrophobic side chains of W4716 from the helical bundle domain between transmembrane helices S2 and S3 (S2S3) and I4996 from CTD, and also stabilized by the hydrogen bonds between one of the two carbonyls and the carboxyl side chain of E4239 from TaF domain (des Georges et al., 2016). These interactions are also conserved in RyR2 (corresponding to RyR2 E4195, W4646, and I4927) (Murayama et al., 2018b). The caffeine-binding site is located directly below the Ca^2+^-binding site. The close proximity of Ca^2+^- and caffeine-binding sites suggests that the two sites play a direct role in regulating Ca^2+^ sensitivity. One of the CPVT mutation, RyR2 W4645R, corresponds to a caffeine-coordinating residues. It not only affects the caffeine binding, but also the structure of the neighboring Ca^2+^-binding site regulating Ca^2+^ sensitivity (Murayama et al., 2018b), which represents a potential mechanism for CPVT.

#### 4.2.2 Chlorantraniliprole

Chlorantraniliprole (CHL), belonging to the diamide insecticide family, is one of the top-selling insecticides on the global market. CHL has a novel mode of action: it can target pest RyRs and cause disruption of feeding and muscle paralysis and ultimately death of the treated insects. One merit of CHL is that it can selectively activate insect RyRs with high nanomolar affinity but binds mammalian RyRs only with a low micromolar affinity (Chen et al., 2019). Recently, the cryo-EM structure of rabbit RyR1 in complex with CHL was solved. It clearly revealed the binding site and the binding pose of CHL at a local resolution of 3.2 Å (Ma et al., 2020). The binding site of CHL was found in a pocket at the interface between the pseudo-voltage-sensor domain (pVSD) from the transmembrane region of RyR and the cytoplasmic core solenoid (CSol) (Figure 6D). The binding of CHL induces a conformational change of pVSD, causing a displacement of the S4-S5 linker to relax the constriction in the S6 bundle and open the pore.

Due to the heavy usage, many mutations have been identified in the insect RyRs, developing the resistance in several agricultural pests, such as diamondback moth (*P. xylostella*) (Nauen and Steinbach, 2016), tomato leafminer (*Tuta absoluta*) (Roditakis et al., 2017), and beet armyworm (*Spodoptera exigua*) (Zuo et al., 2020). The identification of the four resistant mutations (G4946E, I4790M, Y4701D, and Y4922F in DBM numbering) in the transmembrane domain of RyR suggested a potentially nearby diamide-binding pocket, but the exact mechanisms of these resistant mutations remained enigmatic. The RyR1-CHL cryo-EM structure reveals that these four resistance mutations are distributed at the CHL-binding site, but interestingly, they affect CHL binding in two distinct ways: G4946E and I4790M cause steric hindrance with the diamide, while Y4701D and Y4922F reduce the contacts. The Ca^2+^ imaging experiments and insect toxicity results confirm that the mutations cause the resistance in the order of Y4922F→G4946E→Y4701D→I4790M, which agrees with the computational docking results based on the structural model (Ma et al., 2020). The structural basis of diamide modulation of insect RyR provides new insights for the development of diamide derivatives to combat pest resistance.

In addition, as RyR activators, the diamide compounds are also shown to have potential therapeutic effects to treat CCD caused by some loss-of-function (LoF) mutations (Ma et al., 2020). Two cell lines stably expressing LoF CCD mutants, RyR1 R4824C and R4860C, could be activated by CHL with slightly increased EC_50_ values compared to the one expressing WT RyR1, implying that CHL may reduce the LoF effects of these mutations by increasing the opening probability (Ma et al., 2020).

#### 4.2.3 Dantrolene

Dantrolene was used to treat MH and cardiomyopathies effectively by targeting skeletal muscle ryanodine receptors (RyR1) (Paul-Pletzer et al., 2001; Paul-Pletzer et al., 2002) and the cardiac ryanodine receptors (RyR2), respectively (Kobayashi et al., 2009; Uchinoumi et al., 2010). However, the molecular mechanism of dantrolene is mostly unclear. Inter-domain interactions between the N-terminal and central regions of RyRs may be stabilized by binding to the N-terminal residues 590–609 in RyR1 (Paul-Pletzer et al., 2002) and residues 601–620 in RyR2 (Paul-Pletzer et al., 2005). Direct structural evidence supporting these binding sites is missing. According to recent study, dantrolene has no intrinsic effect on purified RyR1 and only shows its effects on the channels pre-activated by other modulators, such as CaM, Mg^2+^, and ATP (Oo et al., 2015; Diszházi et al., 2019).

Wang et al. (2011) used a combination of GFP as a structural marker, FRET, and three-dimensional cryo-EM structures to determine the dantrolene-binding motif in RyR1/RyR2. They proposed that the location of the dantrolene-binding site is located distal to the central region of RyR2 and dantrolene might allosterically modulate the interaction between the N-terminal and central domains. Insertion of GFP after R626, near the proposed dantrolene-binding site, abolished the binding of GST-FKBP12.6, implicating the dantrolene-binding site in close proximity to that for binding FKBP12.6 and the outer periphery of the cytoplasmic assembly (Wang et al., 2011). But the full extent of the functional relationship between these two sites was not investigated. This location was further supported by FRET analyses using 10 FRET pairs (Wang et al., 2011). Further clarification of the dantrolene-regulation on RyR will likely rely on the determination of some high-resolution cryo-EM complex structures or crystal structures of RyR dantrolene-binding domain.

### 4.3 Development of Therapeutic RyR-Targeting Drugs

As a key therapeutic target for many skeletal and cardiac muscle diseases, RyR has recently attracted a lot of attention in drug development. Considering the high structural similarity between RyR1 and RyR2, it is important to develop skeletal or cardiac specific drugs to treat these disease with low side effect. Dantrolene is the only clinically approved drug for MH (Kobayashi et al., 2009). However, it has significant drawbacks in clinical application, including poor water solubility that makes quick preparation difficult under emergency conditions and a long plasma half-life that causes long-lasting side effects such muscle weakness. To tackle this problem, Murayama group identified RyR1-selective inhibitor, oxolinic acid, with improved water solubility using fluoresence-based high-throughput screening (HTS) platform that monitors ER luminal [Ca^2+^] change (Murayama et al., 2018a; Murayama and Kurebayashi, 2019). Following that, they developed a series of oxolinic acid derivatives and successfully developed a compound, 6,7-(methylenedioxy)-1-octyl-4-quinolone-3-carboxylic acid (Cpd1), with comparable potency as dantrolene in *in vitro* study (Mori et al., 2019). This compound effectively rescued mice with MH and heat stroke. Cpd1 has great advantages of higher water solubility and shorter plasma half-life compared to dantrolene, thus representing a promising new candidate drug for the treatment of patients carrying related RyR1 mutations (Yamazawa et al., 2021). In addition, Cornea group developed a series of delicate FRET-based HTS assays to screen isoform-specific inhibitors of RyRs by monitoring the changes of distances between different regulators of RyR, such as CaM, FKBP, DPc10, etc, using which they have identified several promising candidate compounds (Oda et al., 2015; Rebbeck et al., 2017; Rebbeck et al., 2020).

Mutations in RyR2 generally lead to the Ca^2+^ leak that is associated with heart failure and arrhythmias. Current therapy includes indirect inhibition of RyR2 through upstream blockage of β-adrenergic receptors, direct modulation of RyR2, or use of implantable cardioverter defibrillator (ICD). Because β-blockers and ICD devices often fail to prevent potentially lethal arrhythmias, the development of new therapeutic compounds targeting mutant RyR2 is urgently needed (van der Werf et al., 2012). The first compound identified using CPVT mice model was the 1,4-benzothiazepine derivative K201, also known as JTV519, which suppressed ventricular tachycardia caused by SR Ca^2+^ leak (Wehrens et al., 2004a). Subsequently, compound S107, an orally available 1,4-benzothiazepine derivative with high potency on RyR2 and no significant off-target effects, was shown to suppress ventricular arrhythmias in a RyR2 R2474S knock-in mouse model (Lehnart et al., 2008). JTV519 and S107 were proposed to treat CPVT by enhancing the binding of FKBP12.6 to the mutant RyR2 (Wehrens et al., 2004a; Lehnart et al., 2008). Other lead compounds tested for anti-arrhythmic effects in CPVT mouse models include flecainide and carvedilol derivatives (Watanabe et al., 2009; Zhou et al., 2011). But flecainide has some safety problem in patients with structural heart disease (Echt et al., 1991). Knollmann group discovered another antiarrhythmic drug, propafenone, that is effective for CPVT patients through the inhibition of RyR2, similarly to flecainide (Hwang et al., 2011). They also developed ent-1 verticilide, a RyR antagonist derived from a fungal cycloligomeric depsipeptide (Batiste et al., 2019). Compared to those treated with dantrolene, flecainide, and tetracaine, CPVT mice treated with ent-1 verticilide experienced less VT and fewer delayed afterdepolarisations (DADs) (Batiste et al., 2019). Recently, Wehrens group developed tetracaine and its derivatives (EL1-9) for the treatment of ventricular tachycardia (Li et al., 2017). The IC_50_ of EL9 was approximately 400-fold lower than that of JTV519 (Li et al., 2017). Later, a new derivative, known as EL20 [2-(diethylamino)ethyl 4-(butylamino)-2-methoxybenzoate], was discovered and showed similar effect to that of EL9 (Klipp et al., 2018). Furthermore this compound also showed good efficacy against CPVT in human induced pluripotent stem cell-derived cardiomyocytes (iPS-CMs) carrying the R176Q mutation (Word et al., 2021). In addition, Wan et al. 2(2022). reported the discovery that a natural product, Z16b, isolated from *Ganoderma cochlear*, has a potent therapeutic effect on CPVT. It reduces CPVT episodes not only in a mouse model of CPVT but also in iPS-CMs derived from a patient with CPVT. Functional analyses and molecular assays demonstrated that Z16b serves as an RyR2 stabilizer by enhancing the interaction between the CTD and NTD. Given their promising efficacy, the above mentioned compounds represent intriguing therapeutic options, and further development of those compounds may result in a viable therapy for SR Ca^2+^ leak-induced arrhythmia and heart failure. However, the exact binding sites and binding modes of these promising pharmacological compounds agent remain elusive. The determination of high-resolution cryo-EM structures of RyRs in complex with these compounds will clarify their modulatory mechanisms on RyR dysfunctions, thus facilitating the development of isoform-selective potent therapeutic molecules to treat RyR-associated diseases.

## 5 Concluding Remark and Future Prospect

Ryanodine receptors are one of the most complex classes of ion channels, highlighted by their large size and a large number of regulators. The combination of cryo-EM and crystal structures at near-atomic resolution, and biochemical analysis of RyR has provided major insights toward a detailed mechanistic understanding of their function in physiology and pathophysiology. Furthermore, the distribution pattern of mutations in the 3D structure of RyR has been used to generate an efficient tool for diagnosis purpose. Finally, the complex structures of RyR with different modulators not only revealed the positions of several druggable pockets but also provided accurate templates for the rational structure-based drug design. It should be noticed that the present cryo-EM models of RyRs cover only ∼70% of the protein with still ∼1,500 residues missing. Many critical functional domains are missing, including some transmembrane segments, luminal loops, and cytoplasmic fragments. The accurate binding sites of several modulatory small molecules and protein-binding partners are also called into question. Furthermore, the effects of many disease mutations on the full-length RyR structures remain enigmatic, preventing the structure-based rational design of new therapeutics. Determining the structures of recombinant RyRs with disease mutations or PTMs, the super-cluster of RyRs involved in “coupled-gating” and the EC-coupling super-complexes in the absence or presence of a combination of modulators will continue to be a significant focus of future research. The advance in structural biology, such as CLEM and time-resolved cryo-EM, combined with genetic code expansion and long-MD, would shed more light on the challenges in the field.
